# Does Subspecialization Matter? Institutional Outcomes Before and After Recruitment of Colorectal Surgeons

**DOI:** 10.1002/wjs.70519

**Published:** 2026-08-13

**Authors:** Amram Kupietzky, Ata Maden, Roi Dover, Eyal Yonathan Juster, Noam Shussman, Bilal Aliyan, Haggi Mazeh, Ido Mizrahi

**Affiliations:** ^1^ Department of Surgery Hadassah Medical Organization and Faculty of Medicine Hebrew University of Jerusalem Jerusalem Israel

**Keywords:** advanced colorectal surgery, colectomy, colorectal surgeon specialization, colorectal surgery, fellowship, general surgery

## Abstract

**Aim:**

Colorectal surgery fellowship is one of the most sought‐after subspecialty programs among general surgery residents, yet most colon and rectal surgeries are performed by general surgeons without specialized training. This study evaluated whether integrating fellowship‐trained colorectal surgeons (CRS) into a general surgery department is associated with improved outcomes.

**Methods:**

A retrospective cohort study of elective colon and rectal surgeries was conducted at our institution from January 2015 to December 2022. Outcomes before (2015–2017) and after (2018–2022) CRS integration were compared. The primary outcome measured was lymph node yield, an established indicator of oncologic quality while secondary outcomes included operative volume, operative characteristics, postoperative complications, and the length of hospital stay.

**Results:**

Of 586 surgeries included (167 non‐CRS, 419 CRS), baseline demographics were similar. CRS integration was associated with increased operative volume and a higher proportion of complex cases. Laparoscopic surgery increased from 62% to 76% (*p* = 0.09) with a modest rise in conversion to open. Median lymph node harvest improved from 14 to 18 (*p* < 0.01). Time to first defecation decreased from 3 to 2 days (*p* < 0.01); the length of stay and other recovery parameters were comparable. Overall complication and mortality rates did not differ significantly.

**Conclusions:**

Integration of fellowship‐trained colorectal surgeons was associated with increased case volume, higher lymph‐node harvest, greater adoption of minimally invasive techniques, and faster postoperative recovery despite increased case complexity. These findings suggest that subspecialization may enhance departmental performance and patient outcomes, although the potential impact of concurrent practice changes cannot be excluded.

## Graphical abstract

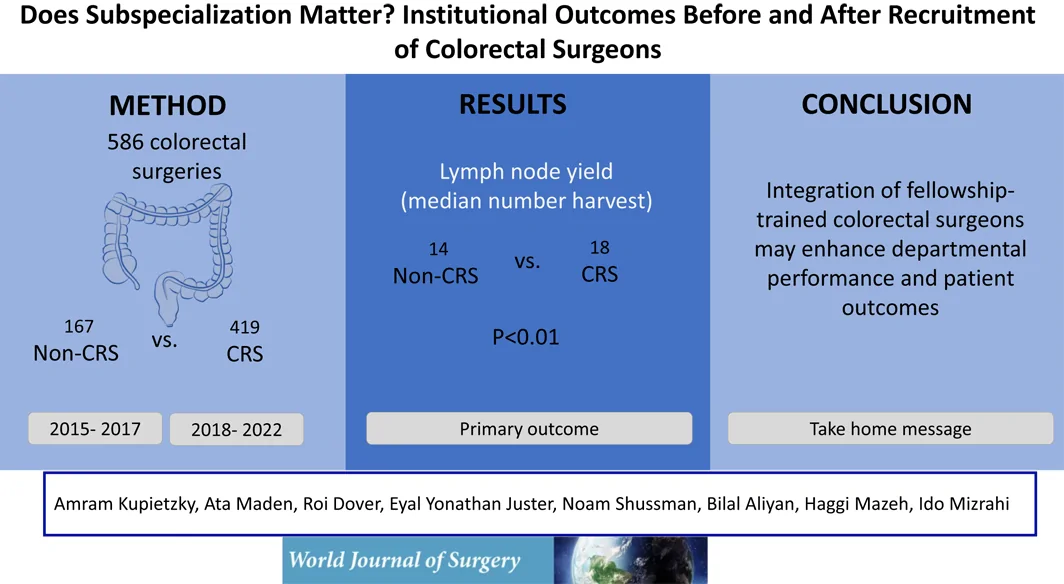

## 1 Introduction

Colorectal cancer (CRC) is the third most diagnosed malignancy in the United States and the third leading cause of cancer‐related mortality [1]. Although the management of CRC is multidisciplinary, approximately two‐thirds of patients ultimately require major surgery, as oncologic resection remains the cornerstone of curative treatment and a critical determinant of long‐term survival [2]. Correspondingly, colorectal fellowship training has become one of the most sought‐after subspecialties among general surgery residents [3]. Despite this trend, the majority of colorectal procedures in the United States are still performed by general surgeons without subspecialty certification, and fewer than one‐quarter are carried out by fellowship‐trained colorectal surgeons [4].

Whether the involvement of colorectal surgeons (CRS) translates into improved surgical quality or patient outcomes remains uncertain. Existing literature is limited, heterogeneous, and often constrained by small cohorts or single‐pathology analyses [5, 6].

In December 2017, two surgeons who had completed formal colorectal fellowship training joined our general surgery department, creating a natural experiment to assess the effect of CRS integration into routine clinical practice. To note, our department did not have a CRS before their arrival. This study aimed to evaluate the impact of CRS on outcomes of colorectal surgery performed within a general surgery department. The primary outcome was lymph node yield an established indicator of oncologic quality while secondary outcomes included operative volume, operative characteristics, postoperative complications, and length of hospital stay.

By examining a multi‐year experience before and after CRS integration, this study provides real‐world evidence on the impact of subspecialty‐trained colorectal surgeons on surgical quality and patient outcomes, addressing an important gap in the current literature.

## 2 Materials and Methods

### 2.1 Study Design

Following the approval of our institutional review board, a retrospective cohort study was conducted to identify all patients who underwent elective colon and rectal surgery between January 1, 2015, and December 31, 2022, in our general surgery department. Emergent cases and procedures performed within 30 days of a prior abdominal surgery were excluded to avoid confounding from acute pathology or repeat interventions.

Patients were categorized into two periods: the Non‐CRS period (January 2015–December 2017), during which no post‐fellowship colorectal surgeon was present. The second period was the CRS period (January 1, 2018–December 31, 2022), after two fellowship trained colorectal surgeons joined the surgical team.

### 2.2 Data Collection

Data were extracted from electronic medical records, including patient demographics (age, sex, BMI, ASA score, comorbidities) and operative characteristics (procedure type and indication, surgical approach, operative and anesthesia duration, resection type, colostomy creation and etc). Postoperative outcomes included time to first defecation, time to urinary catheter removal, complications graded by the Clavien–Dindo classification (minor: grades I–II; major: grades III–IV), length of stay, readmission, and mortality. Pathology results were abstracted from expert pathologist reports.

### 2.3 Surgical Indications

Surgical indications were grouped into malignancy, benign tumors, inflammatory bowel disease (IBD), diverticulitis, volvulus, advanced colorectal (ACR), and other. ACR procedures included *trans*‐anal minimally invasive surgery (TAMIS), perineal rectosigmoidectomy, *trans*‐anal excision (TAE), and proctocolectomy with ileal pouch–anal anastomosis (J‐pouch). The “other” category included operations for complicated endometriosis with bowel involvement, recurrent inguinal hernia with bowel fistula, and colostomy closure (“Hartmann closure”).

### 2.4 Statistical Analysis

Baseline demographic variables, operative characteristics, and surgical indications were compared between cohorts. Continuous variables were assessed for normality and compared using independent‐samples t‐tests or Mann–Whitney U tests. Categorical variables were analyzed using Chi‐square. Fisher's exact test was applied when expected cell counts were < 5. Complications were compared after stratifying into minor and major categories.

To account for differing period lengths (3 vs. 5 years), operative volumes, surgical indications, and complication frequencies were analyzed both as absolute totals and normalized to cases per year. A two‐sided *p*‐value < 0.05 was considered statistically significant. Analyses were performed using SPSS version 26 (SPSS Inc.). Results are presented as mean ± standard deviation or median (interquartile range), as appropriate.

## 3 Results

**FIGURE 1:**
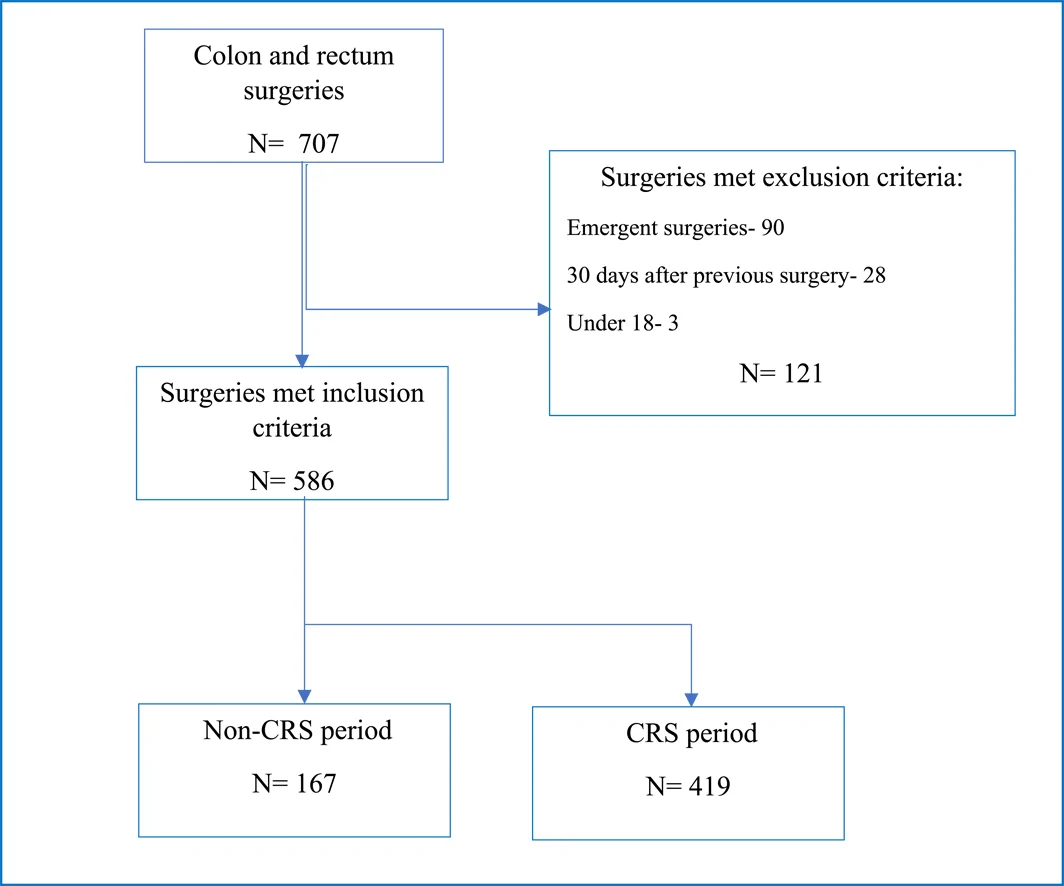
Inclusion algorithm.

During the study period, 707 colon and rectum surgeries were performed in our department. After applying exclusion criteria (emergent surgery, age < 18 years, or surgery within 30 days), 586 patients were included in the final analysis (Figure 1). Of these, 167 underwent surgery during the non‐CRS period and 419 during the CRS period.

The two cohorts were comparable in baseline characteristics, including age, sex distribution, BMI, and ASA score (Table 1).

**TABLE 1:** Patient demographics.

	Non‐CRS period *N* = 167	CRS period *N* = 419	*p* value
Age, median (IQR)	65 (55–78)	64 (52–73)	0.26
Male gender	79 (47.3%)	208 (49.6%)	0.61
BMI, median (IQR)	26.6 (22.7–30)	26.08 (23.2–30)	0.54
ASA score (mean)	2.08 ± 0.68	2.29 ± 0.72	0.43

Abbreviation: BMI: Body mass index.

The CRS period was associated with a marked increase in operative volume (55 cases per year vs. 84 cases per year, *p* < 0.001), driven by a shift toward more complex elective colorectal cases. Elective sigmoidectomy for diverticulitis (*n* = 18, 6.2%) and advanced colorectal surgeries (*n* = 23, 5.5%), were performed only during the CRS period. The annual rate of oncologic resections increased from 45.3 cases per year during the 3‐year non‐CRS period (136 surgeries) to 54.2 cases per year during the 5‐year CRS period (271 surgeries). In parallel, the proportion of surgeries performed for malignancy decreased from 81.4% to 64.7% (*p* = 0.03). The surgical approach differed significantly between periods. Laparoscopic surgery increased from 62.2% to 75.8% (*p* = 0.086), while open surgery decreased from 37.1% to 20.5% (*p* = 0.03). Conversion to open surgery was more frequent during the CRS period (3.5% vs. 0.6%, *p* = 0.07). Anesthesia time was similar between groups (258 (207–320) versus. 240 (190–320) min, median (IQR), *p* = 0.12), as were rates of stoma formation (10.2% vs. 13.8%, *p* = 0.22) and intraoperative blood transfusion (17.3% vs. 12.6%, *p* = 0.14) (Table 2).

**TABLE 2:** Operative data.

		Non‐CRS period *N* = 167	CRS period *N* = 419	*p* value
Indication	Malignancy	136 (81.4%)	271 (64.7%)	0.03
	Benign tumor	6 (3.6%)	34 (8.8%)	0.29
	IBD	5 (3.0%)	9 (4.3%)	0.75
	Diverticulitis	0 (0%)	18 (6.2%)	0.0003
	Volvulus	6 (3.6%)	26 (3.3%)	0.48
	Advanced colorectal*	0 (0%)	23 (5.5%)	0.0001
	Others	14 (8.4%)	28 (6.7%)	0.93
Approach	Laparoscopic	104 (62.2%)	318 (75.8%)	0.086
	Open	62 (37.1%)	86 (20.5%)	0.03
	Conversion to open (%)	1 (0.6%)	15 (3.5%)	0.07
Anesthesia time, median (IQR)		258 (207–320)	240 (190–320)	0.12
Stoma		17 (10.2%)	58 (13.8%)	0.22
Blood transfusion		29 (17.3%)	53 (12.6%)	0.14

Abbreviation: IBD‐ Inflammatory bowel disease.

*Advanced colorectal‐trans‐anal minimally invasive surgery, perineal rectosigmoidectomy, *trans*‐anal excision, and proctocolectomy with ileal pouch–anal anastomosis (J‐pouch).

Oncologic quality metrics improved substantially during the CRS period. The median number of lymph nodes harvested increased from 14 (IQR 8–20) to 18 (IQR 10–30), with mean values rising from 14.18 to 21.3 nodes (*p* < 0.01). The number of positive lymph nodes was similar between groups (1.53 vs. 1.54, *p* = 0.98) (Table 3).

**TABLE 3:** Post‐operative data.

	Non‐CRS period *N* = 167	CRS period *N* = 419	*p* value	Or (CI)
Time to defecation, median (IQR)	3 (3–4)	2 (2–3)	< 0.01	
Time to catheter excision (days)	2 (1–3)	1 (1–2)	0.10	
Hospitalization days after operation (days)	6.45 ± 3.4	6.27±	0.8	
Lymph nodes in pathology^a^	14.18	21.3	< 0.01	
Lymph nodes in pathology, median (IQR)^a^	14 (8–20)	18 (10–30)		
Affected lymph nodes in pathology^a^	1.53	1.54	0.98	
Average clavien dindo (mean)	0.5	0.7	0.06	
Complication	37 (22%)	147 (35%)	0.10	
Prolonged ileus	2 (1.1%)	22 (5.3%)	0.15	
UTI	1 (0.6%)	6 (1.4%)	0.09	
Pneumonia	3 (1.7%)	7 (1.7%)	0.33	
Atelectasis	1 (0.6%)	4 (1.4%)	0.66	
Surgical site infection	12 (7.2%)	18 (4.3%)	0.19	
CVA	1 (0.6%)	2 (0.48%)	1.0	
Arrhythmia	1 (0.6%)	10 (2.4%)	0.3	
Leakage	8 (4.8%)	15 (3.6%)	0.51	
Abdominal abscess	2 (1.2%)	15 (3.6%)	0.38	
Bleeding	7 (4.2%)	12 (2.9%)	0.44	
Minor complications	27 (16.2%)	112 (26.7%)	0.007	1.89 (1.19–3)
Major complications	7 (4.2%)	38 (9.1%)	0.046	2.28 (1–5.2)
Mortality	2 (1.2%	1 (0.24%)	0.2	0.2 (0.02–2)
Readmission in 30 days	8 (4.8%)	36 (8.6%)	0.12	1.87 (0.86–4.05)

Abbreviations: CVA, cerebral vascular accident; UTI, urinary tract infection.

^a^ Excluding surgeries with a primary indication of IBD, diverticulitis and volvulus.

Postoperative recovery parameters analysis demonstrated an earlier return of bowel function during the CRS period: time to first defecation decreased from a median of 3 days (IQR 3–4) to 2 days (IQR 2–3) (*p* < 0.01). However, time to urinary catheter removal and postoperative length of stay did not differ between groups.

A trend toward a higher overall complication rate during the CRS period (35% vs. 22%) was observed, although this difference did not reach statistical significance (*p* = 0.10). Minor complications were more frequent (26.7% vs. 16.2%, OR 1.89, 95% CI 1.19–3.0), as were major complications (9.1% vs. 4.2%, OR 2.28, 95% CI 1.0–5.2). Rates of specific complications, including anastomotic leak, pneumonia, urinary tract infection, abdominal abscess, and bleeding, were similar between groups. Mortality was low in both periods (1.2% vs. 0.24%, *p* = 0.20), with no statistically significant difference. Readmission within 30 days occurred in 4.8% of patients in the non‐CRS period and 8.6% in the CRS period (*p* = 0.12) (Table 3).

## 4 Discussion

The integration of CRS into general surgery departments is an increasingly relevant issue as sub specialization becomes standard in surgical training [7, 8]. In this retrospective cohort study, the presence of CRS was associated with improvement in oncologic quality metrics, most notably lymph node harvest, increased case volume, faster recovery of bowel function, and a clear shift toward advanced and complex colorectal surgery. These findings suggest that subspecialty expertise may improve technical, clinical, and pathologic aspects of patient care, in a general surgery ward setting.

One of the observed findings was the significant increase in lymph node harvest during oncologic resections following CRS integration. Lymph node yield is a widely used surrogate marker of oncologic quality and has been used in prior research as an indicator of adequacy of oncologic resection and staging quality, although it is recognized to be influenced by both surgical technique and pathological assessment [9]. Prior studies have reported higher lymph node retrieval rates among surgeons with dedicated colorectal training compared with general surgeons, which may reflect greater familiarity with mesenteric anatomy and heightened attention to oncologic principles [10, 11, 12]. Consistent with these reports, we observed an increase in mean lymph node yield from 14.1 to 22.3. In our institution, specimens were processed within the same pathology department throughout the study period, and no major changes in reporting standards or grossing protocols were identified, although subtle variability in pathological assessment over time cannot be fully excluded.

Although the harvest of at least 12 lymph nodes is traditionally recommended for adequate staging, accumulating evidence suggests that higher thresholds, ranging from 18 to 24 nodes, are associated with improved oncologic outcomes [13, 14, 15, 16]. Ning et al. identified the harvest of 18 lymph nodes as an optimal benchmark for improved survival [17]. Notably, the mean lymph node yield achieved during the CRS period met this proposed threshold, reinforcing the association between colorectal subspecialty training and enhanced oncologic quality of resection.

Beyond oncologic metrics, postoperative recovery parameters also improved during the CRS period. Time to first bowel movement decreased significantly, and length of stay was modestly reduced. A similar reduction has been reported by Saraidaridis et al., who found that length of stay was 0.8 days shorter among patients undergoing surgery performed by CRS [18]. These improvements occurred despite a higher proportion of complex procedures, including advanced colorectal operations, elective diverticulitis resections, and an increased IBD case volume, suggesting that the presence of CRS may contribute to improved perioperative decision‐making, greater standardization of care pathways, and more consistent postoperative management across the department.

The observed increase in postoperative complications during the CRS period warrants careful interpretation. While both minor and major complications were more frequent, this trend likely reflects the increased complexity of cases undertaken after CRS integration rather than a decline in surgical quality. The introduction of advanced procedures, including elective surgery for sigmoid diverticulitis, perineal rectosigmoidectomy, and complex IBD operations, inherently carries higher baseline risk profiles. Importantly, mortality remained low and did not differ significantly between periods, reinforcing that overall procedural safety was preserved.

This interpretation is supported by prior literature demonstrating favorable outcomes associated with colorectal subspecialty training. In a recent nationwide analysis, Purdy et al. reported significantly lower rates of postoperative complications and in‐hospital mortality for colectomies and proctectomies performed by colorectal surgeons compared with general surgeons, even after adjustment for case complexity [6]. Similar findings have been described by Saraidaridis et al., further suggesting that colorectal subspecialty training may mitigate operative risk in complex colorectal procedures [18]. Taken together, these findings suggest that the higher complication rates observed during the CRS period are more likely related to increased case complexity rather than a decline in surgical quality.

The use of minimally invasive surgery increased during the CRS period (62.2% vs. 78.5%, *p* = 0.086). Although this difference did not reach conventional statistical significance, it is consistent with broader trends toward increased adoption of laparoscopic colorectal surgery over time and with the higher baseline laparoscopic exposure typically associated with colorectal fellowship training [3, 19]. This increase in minimally invasive approach was accompanied by a higher conversion rate. Taken together, these findings likely reflect a combination of increasing case complexity during the CRS period and the expanded application of minimally invasive techniques to more challenging cases, with conversion appropriately employed when required for patient safety.

Beyond clinical outcomes, the integration of CRS had meaningful educational implications for the general surgery residency. The CRS period introduced greater operative complexity, partly due to the addition of advanced colorectal procedures (23 cases [5.5%]), providing residents with exposure to operations rarely encountered in standard training, such as TAMIS, perineal rectosigmoidectomy, and complex pelvic dissections. These cases support the development of refined technical skills and complex intraoperative decision‐making. In parallel, the establishment of structured multidisciplinary team (MDT) meetings for colorectal oncology and IBD created additional learning opportunities by engaging residents in evidence‐based discussions on staging, treatment planning, and postoperative management. The presence of fellowship‐trained colorectal surgeons also fostered a more academically active environment, reflected in increased participation in research, case discussions, and departmental publications. As surgical education continues to shift toward competency‐based training and readiness for independent practice, these findings underscore the educational value of incorporating subspecialty‐trained surgeons into general surgery departments.

Although economic outcomes were not directly assessed in this current investigation, the implications are noteworthy. Prior literature suggests that colorectal fellowship training may be associated with lower individual income trajectories compared with general surgery practice [20, 21]. However, at the departmental level, factors such as increased operative volume, expansion of surgical indications, shorter hospital length of stay, and improved oncologic quality metrics may translate into enhanced productivity and potential financial benefit for the institution. Further investigation is warranted to formally evaluate the economic impact of CRS integration, as these considerations may be particularly relevant for departmental leadership and institutional decision‐makers.

This study has limitations inherent to its retrospective design. The comparison of two distinct time periods introduces the potential for historical bias. During the study period, advances in colorectal cancer care, including increased adoption of minimally invasive surgery, implementation of enhanced recovery pathways, and broader improvements in perioperative management, may have influenced outcomes independently of subspecialization. Conversely, some of these developments may have been facilitated by the integration of fellowship‐trained colorectal surgeons, making it difficult to fully disentangle the effects of subspecialization from contemporary changes in clinical practice. Nevertheless, no major institutional changes in pathology processing or formal perioperative care protocols occurred during the study period other than the integration of fellowship‐trained colorectal surgeons.

Residual confounding related to case mix, disease severity, and other unmeasured clinical factors cannot be fully excluded. Operator‐related bias is unavoidable in surgical outcomes research, however, the stability of the surgical team and the clear timing of CRS integration support the internal validity of our findings. Importantly, more robust oncologic quality measures, including TME specimen quality, CME grading, circumferential resection margin status, and local recurrence, were not consistently available in this retrospective dataset and therefore could not be analyzed. Accordingly, lymph node yield and perioperative outcomes were used as surrogate endpoints. Finally, as a single‐center study, the generalizability of these findings may be limited, particularly to institutions with different case volumes, staffing models, or referral patterns.

## 5 Conclusion

The integration of fellowship‐trained colorectal surgeons into a general surgery department was associated with measurable changes in colorectal surgery care, including increased lymph node harvest, enhanced postoperative recovery, and management of a more complex surgical case mix. Although some findings may reflect broader evolution in colorectal care over time, these changes are consistent with the potential benefits of colorectal subspecialization within a general surgery department. Our findings suggest that integration of specialized surgeons may contribute to improvements in departmental performance and the delivery of colorectal surgery care. These results should be interpreted in the context of a retrospective, single‐center study, and further prospective or multi‐institutional studies are warranted to better define the independent impact of colorectal subspecialization. Nevertheless, these findings may be of relevance to institutions and policy makers when evaluating the role and allocation of advanced colorectal surgery training within modern surgical practice.

## Author Contributions

**Amram Kupietzky:** conceptualization, investigation, writing–original draft, methodology, validation, visualization, writing–review and editing, formal analysis, project administration, data curation, supervision, resources. **Roi Dover:** investigation, visualization, formal analysis, data curation. **Ata Maden:** investigation, formal analysis, visualization, data curation. **Bilal Aliyan:** methodology, formal analysis, supervision, visualization. **Ido Mizrahi:** conceptualization, investigation, writing–original draft, methodology, validation, visualization, writing–review and editing, formal analysis, project administration, supervision. **Haggi Mazeh:** conceptualization, investigation, writing–review and editing, supervision. **Noam Shussman:** conceptualization, writing–review and editing, methodology, supervision. **Eyal Yonathan Juster:** investigation, validation, formal analysis, data curation.

## Funding

The authors have nothing to report.

## Disclosure

The authors have nothing to report.

## Ethics Statement

This study was conducted in accordance with institutional and international ethical standards. Ethics approval was obtained from the Hadassah Medical Center Institutional Review Board (Approval ID: HMO‐0766‐20).

## Consent

The requirement for informed consent was waived by the ethics committee due to the use of de‐identified data.

## Conflicts of Interest

The authors declare no conflicts of interest.

## Data Availability

The data that support the findings of this study are available on request from the corresponding author. The data are not publicly available due to privacy or ethical restrictions.
